# Rational design of allosteric switchable catalysts

**DOI:** 10.1002/EXP.20210095

**Published:** 2022-02-23

**Authors:** Tiezheng Pan, Yaling Wang, Xue Xue, Chunqiu Zhang

**Affiliations:** ^1^ State Key Laboratory of Medicinal Chemical Biology Nankai University Tianjin China; ^2^ School of Life Sciences Northwestern Polytechnical University Xi'an China

**Keywords:** allosterism, molecular machine, self‐assembly, supramolecular catalysis, switchable catalyst

## Abstract

Allosteric regulation, in many cases, involves switching the activities of natural enzymes, which further affects the enzymatic network and cell signaling in the living systems. The research on the construction of allosteric switchable catalysts has attracted broad interests, aiming to control the progress and asymmetry of catalytic reactions, expand the chemical biology toolbox, substitute unstable natural enzymes in the biological detection and biosensors, and fabricate the biomimetic cascade reactions. Thus, in this review, we summarize the recent outstanding works in switchable catalysts based on the allosterism of single molecules, supramolecular complexes, and self‐assemblies. The concept of allosterism was extended from natural proteins to polymers, organic molecules, and supramolecular systems. In terms of the difference between these building scaffolds, a variety of design methods that tailor biological and synthetic molecules into controllable catalysts were introduced with emphasis.

## Graphical abstract

Allosteric regulation controls the activities of natural enzymes and expands the chemical biology toolbox for the research on switchable catalysts. These rationally designed substitutes for unstable natural enzymes have been applied in areas such as biosensors and biomimetic cascade reactions. This review extended the concept of allosterism to artificial scaffolds and introduced a variety of design methods for controllable catalysts.

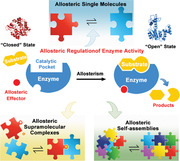

## 1 INTRODUCTION

Billions of years of evolution allow organisms to possess a variety of natural enzymes that can control the progress of biochemical reactions with incredible substrate specificity and activity under physiological conditions. Therefore, it is of broad interest to learn from nature's masterpieces and develop bioinspired enzymes for promising applications in biosensors,^[^ ^1^, ^2^, ^3^ ^]^ pollutant removal,^[^ ^4^, ^5^, ^6^ ^]^ and organic synthesis.^[^ ^7^, ^8^, ^9^ ^]^ From the global energy issue to human health, these human‐made catalysts have offered new solutions to benefit humankind with nature's wisdom. For instance, the alarming fact that currently most of the global energy supply is generated from non‐renewable carbon‐based fuels has concerned the sustainable development of humanity, while mimicking hydrogenase and splitting of water into molecular hydrogen with solar energy might be a promising way to solve the world's energy issue.^[^ ^10^, ^11^, ^12^ ^]^ Also, the respiration process in organisms not only provides energy but also could cause cardiovascular disease, which is one of the biggest killers to humans. Artificial peroxidases would relieve the oxidative pressure from respiration and postpone the aging process.^[^ ^13^ ^]^ Compared with traditional synthetic catalysts, the study of biocatalysts tends to focus on the design of enzyme‐like catalytic mechanisms and the development of biological production technologies. Recently, versatile molecular scaffolds were utilized to afford exceptional examples aiming at natural enzymes’ expertise: facilitating the formation of enzyme‐substrate complex and reducing the activation energy barrier of catalytic reactions. Essential knowledge and critical factors (e.g., hydrogen bonds, electrostatic potential, van der Waals’ forces, coordination bonds) that govern the enzyme activity have been explored by studying different models such as small organic molecules,^[^ ^14^, ^15^, ^16^, ^17^, ^18^, ^19^, ^20^ ^]^ polymers,^[^ ^21^, ^22^, ^23^, ^24^, ^25^, ^26^, ^27^, ^28^, ^29^ ^]^ supramolecular complexes,^[^ ^30^, ^31^, ^32^, ^33^, ^34^, ^35^, ^36^, ^37^ ^]^ natural proteins,^[^ ^38^, ^39^, ^40^, ^41^, ^42^, ^43^, ^44^ ^]^ and protein assemblies.^[^ ^45^, ^46^, ^47^, ^48^, ^49^, ^50^, ^51^, ^52^ ^]^ These multidisciplinary works have spawned many successful bioinspired enzymes, some of which even showed high efficiencies comparable to their natural counterparts.

Besides high catalytic efficiencies, regulation mechanisms of natural enzymes are also critical. The biosynthesis and consumption of saccharides, lipids, and amino acids are all regulated by turning on/off the key enzymes in the specific metabolic pathways. Also, the homeostasis in organisms must be maintained by the control of enzyme activities to ensure the stable concentrations of substances under normal physiological conditions. For instance, taking overdosed high‐efficiency antioxidant agents to inhibit ROS damage would inevitably lead to disorders of the body's cellular signal transduction systems, which is not conducive to normal metabolism.^[^ ^53^ ^]^ In this case, the ideal artificial enzymes must not only show high activity but also be well regulated to perform their functions intelligently. On the other hand, the study of cascade reactions and industrial productions needs the development of controllable catalysts to manipulate the reaction progress for both academic research and practical application. Hence, it is of great value to mimic living systems through the design of switchable bioinspired enzymes by changing their structure and properties in response to external stimuli.

**SCHEME 1:**
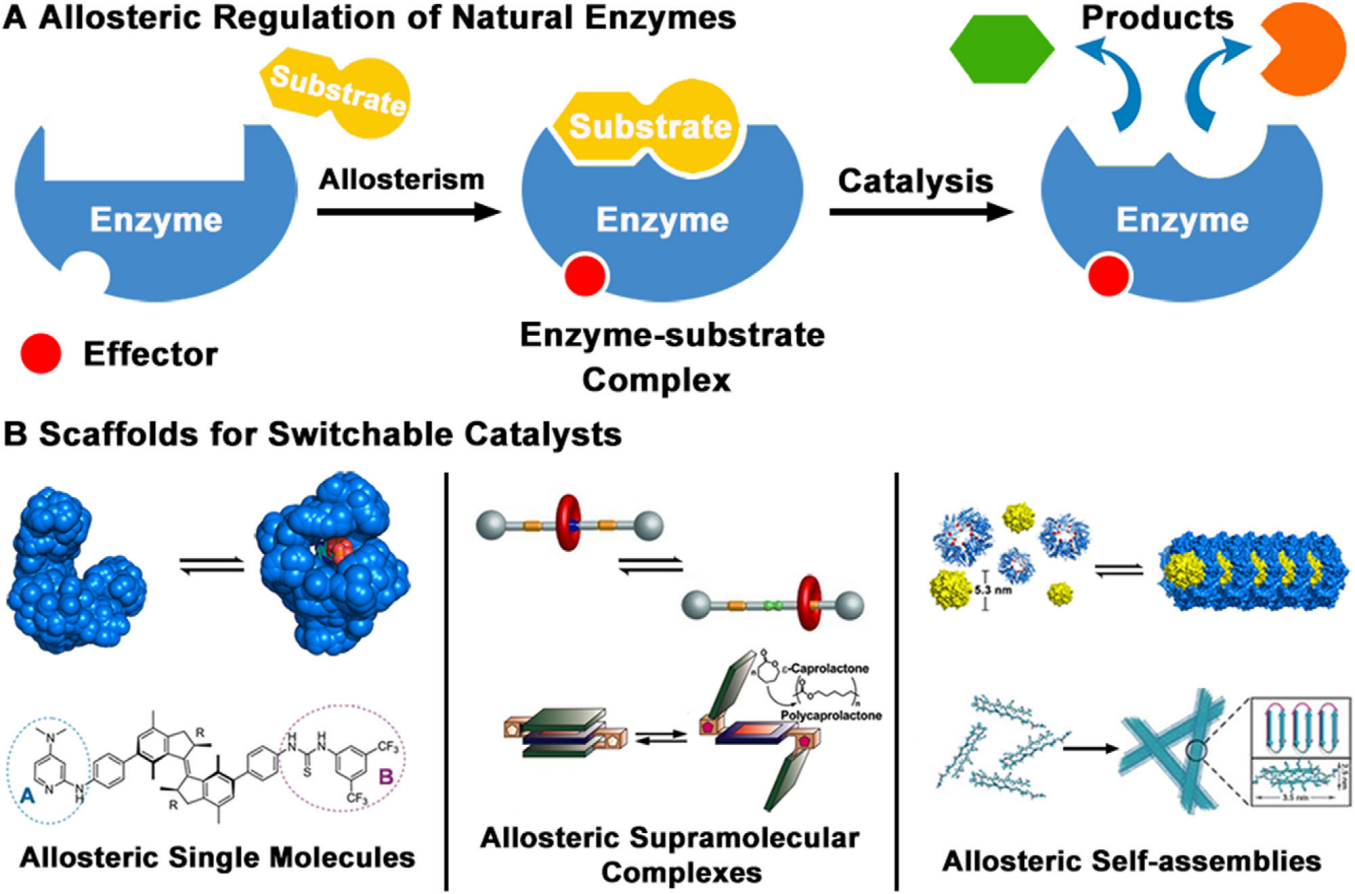
(A) The activity of natural enzymes is strictly controlled by allosteric regulation. (B) Artificial switchable catalysts have been designed by incorporating catalytically active groups in allosteric scaffolds

To facilitate the control of catalytic activities, natural enzymes have acquired the ability of allosteric regulation through evolution (Scheme 1A). The catalytic activity of natural enzymes could be turned on/off by binding an “effector” molecule which conducts the conformational change of the enzyme (allosterism). This effector‐binding site is usually remote from the catalytic site but still able to significantly affect the catalytic behavior. The presence and amount of the allosteric effector in the cell could be a specific indicator to observe the metabolic pathway in which the enzyme is involved. In this way, the catalytic activity of the enzyme, as well as the metabolic pathway, could be regulated. Inspired by natural enzymes, it is critical to combine the catalyst design strategies with a variety of stimulus‐responsive materials, bringing the characteristics of sensitivity to external stimuli such as temperature, pH, ionic strength.^[^ ^54^ ^]^ With the vigorous development and wide application of stimulus‐responsive materials,^[^ ^55^ ^]^ molecular machines,^[^ ^56^ ^]^ protein/peptide assemblies,^[^ ^57^ ^]^ and many other scaffolds have been discovered. Generally, these reversible processes control the on/off regulation of enzymes by changing the conformations of the enzyme scaffolds to reveal/embed the catalytic sites upon specific conditions.

With the development of supramolecular chemistry and catalytic technology, many controllable catalysts based on synthetic molecular switches have been highlighted.^[^ ^58^ ^]^ Compared to previous reports on this topic, this review broadened the notion of “allosteric effect” and run through a variety of catalyst‐design ideas which apply to both synthetic molecules and biomolecules. Typically, the inherent allosteric behavior of natural proteins could be utilized as universal building scaffolds for switchable protein catalysts. Recent advances in the field of bioinspired switchable catalysts designed by allosteric molecules, supramolecular complexes, and self‐assemblies are introduced (Scheme 1B). Artificially designed proteins, peptides, and synthetic molecules with high catalytic activities and defined regulatory mechanisms have been summarized. Instead of a comprehensive introduction, typical examples are presented with an emphasis on the design strategy and catalytic behavior. We expect these contents would provide insight into the construction of bioinspired switchable catalysts for a wide range of applications.

## 2 ALLOSTERIC CATALYSTS BASED ON SINGLE MOLECULES

At the molecular level, the allosteric effect has been mostly considered as the change of substrate‐binding capacity. Typically, the allosteric proteins coordinate with the effector molecule at the first binding site, which changes the substrate binding at the second binding site. This remote control from the first to the second binding site is transferred through the conformational change across the protein. For the design of artificial switchable catalysts based on allosteric effects, it is critical to build the conformational or geometrical change between the regulatory site and catalytic site. On the other hand, current achievements in this field mostly utilize another strategy: incorporating the catalytic group into existing allosteric scaffolds such as naturally occurring allosteric proteins and synthetic stimuli‐responsive molecules.

### 2.1 Switchable protein‐based catalysts

With the development of computer performance and design algorithms, the construction and evaluation of bioinspired switchable catalysts could be performed in silico to provide an essential reference in practice. The rational design of proteins and peptides has been widely studied to acquire specific structures and functions based on the existing knowledge of natural enzymes, such as crystal structures and catalytic mechanisms. Specifically, this idea of computer‐aided design was proved efficient in the engineering or redesign of natural enzymes and their homologous family members, as minimal modifications on the active sites could yield novel catalytic performance.^[^ ^59^ ^]^ As the innate feature of the protein scaffold must serve the design purpose of the enzyme, designing a switchable enzyme is usually accompanied by allosteric protein scaffolds with naturally occurring controllability.

For the design to start with, the allosteric protein scaffold must be able to bind the substrate to form the enzyme‐substrate complex in the “on” conformation, while avoiding the substrate binding in the “off” conformation. Many automated software/servers such as AutoDock, DOCK, ZDOCK, SwarmDock, etc., have been developed for molecular docking. The computed results of these algorithms predict the preferred orientation and the binding free energy, which are crucial information for enzyme design. Second, genetic engineering or chemical modification procedures will be utilized to endow the protein scaffold with catalytic function, meanwhile, the conformation of the scaffold should remain stable without dramatic structural fluctuation. In this regard, molecular dynamic programs such as GROMACS, AMBER, CHARMM, etc. provide comprehensive sets of simulation, analysis, and model building tools to verify the enzyme design. Specifically, the computed averaged root mean square deviation of the modified protein scaffold has a reference value on the system stability, and the variables of the hydrogen bonding, binding free energy, and electrostatic potential distribution all reveal advantages and disadvantages of the current design. In addition, the activities of these computationally designed biocatalysts could be further improved by directed evolution. Starting from the pioneering studies by Stemmer,^[^ ^60^, ^61^ ^]^ Arnold,^[^ ^62^ ^]^ Reetz,^[^ ^63^ ^]^ and Bornscheuer,^[^ ^64^ ^]^ many problems of bioinspired enzymes such as stability and limited substrate scopes have been solved by directed evolution. These two inseparable paths can serve complementary roles in novel enzyme design.

Scrimin and co‐workers built a metal ion regulated peptide template as an allosteric supramolecular catalyst for the cleavage of phosphate esters.^[^ ^65^ ^]^ The target tripodal peptide [T(P1)_3_] was obtained based on the helix heptapeptide template (P1a) reported previously^[^ ^66^ ^]^ by connecting three copies of P1a to a functionalized Tris(2‐aminoethyl)amine (Tren) platform. [T(P1)_3_] could bind up to four metal ions (Cu^II^ or Zn^II^): one in the Tren subsite and three in the azacyclononane subunits. The binding of the metals to the Tren platform would induce a change in which the three short, helical peptides are aligned in a parallel manner with the azacyclonane units pointing inward within the pseudocavity they define. Thus, the activity of the catalyst could be controlled in opposite ways by Zn^II^ according to the type of substrate used. In the cases of the 2‐hydroxypropyl‐*p*‐nitrophenyl phosphate (HPNP) cleavage, Zn^II^ could switch on the activity as a positive allosteric regulator. For an oligomeric RNA substrate, however, Zn^II^ switches off the activity as a negative allosteric regulator. This tripodal peptide‐based supramolecular catalyst provides a striking example of the selective mode of action toward two different phosphate esters and shows how, by increasing the complexity of a supramolecular catalyst.

**FIGURE 1:**
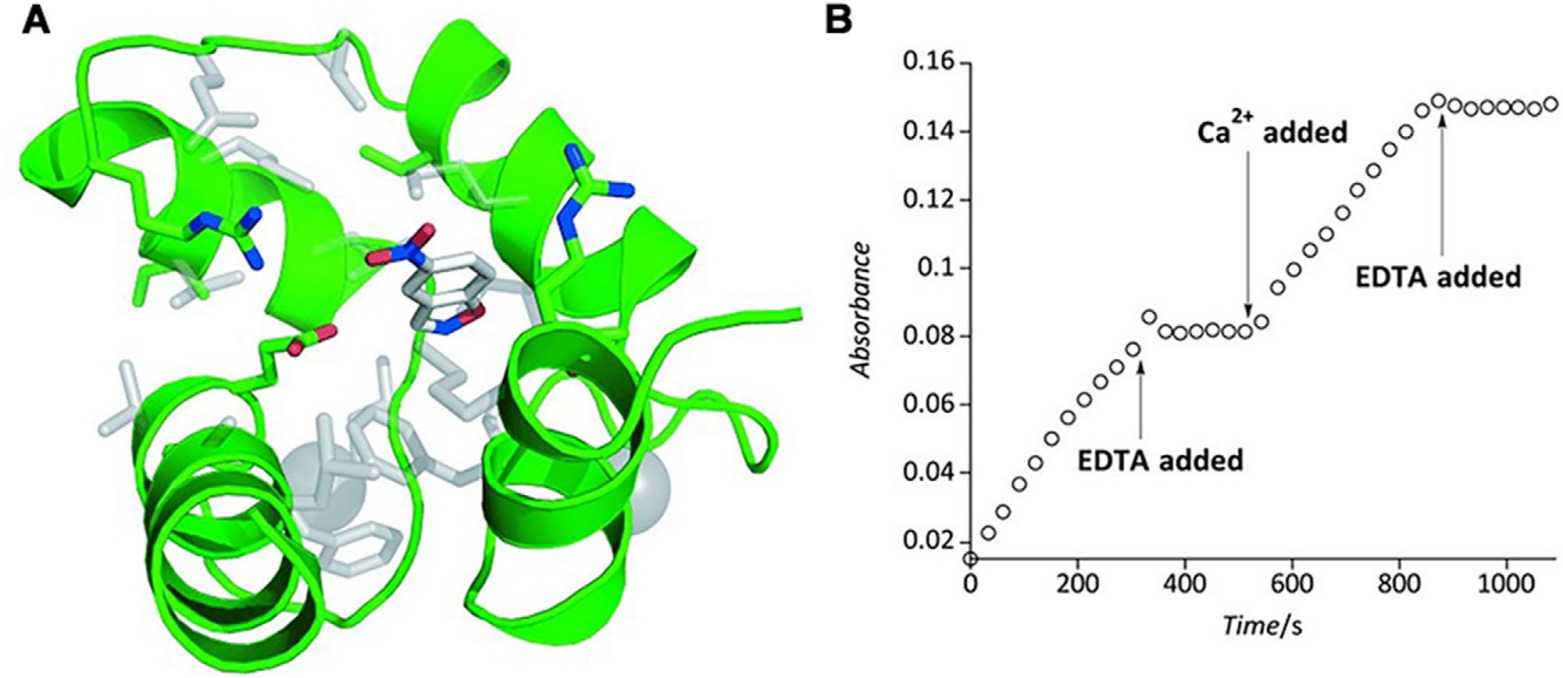
(A) NMR structure of the CaM F92E. (B) Allosteric regulation of the CaM based catalyst. Adapted with permission.^[^ ^68^ ^]^ Copyright 2011, Wiley‐VCH

The allosteric protein calmodulin (CaM) has been chosen as a scaffold because CaM would form a hydrophobic environment upon Ca^2+^ binding. By computational design and the incorporation of a carboxylate residue into the hydrophobic pocket of CaM (F92E) (Figure 1), it showed significant Kemp eliminase activity with a *k*_cat_/*K*_M_ value of 6 M^–1^ s^–1^, which is within an order of magnitude of those of the most active artificial Kemp eliminases.^[^ ^67^ ^]^ More importantly, the engineered CaM exhibited excellent allosteric regulation that no activity was observed in the absence of Ca^2+^. The subsequent directed evolution procedure further improved the *k*_cat_/*K*_M_ value by more than 200‐fold without affecting the regulatory efficiency.^[^ ^68^ ^]^ This result proved that minimal design such as one single mutation at the proper position could afford an efficient artificial enzyme from a simple scaffold without activity.

**FIGURE 2:**
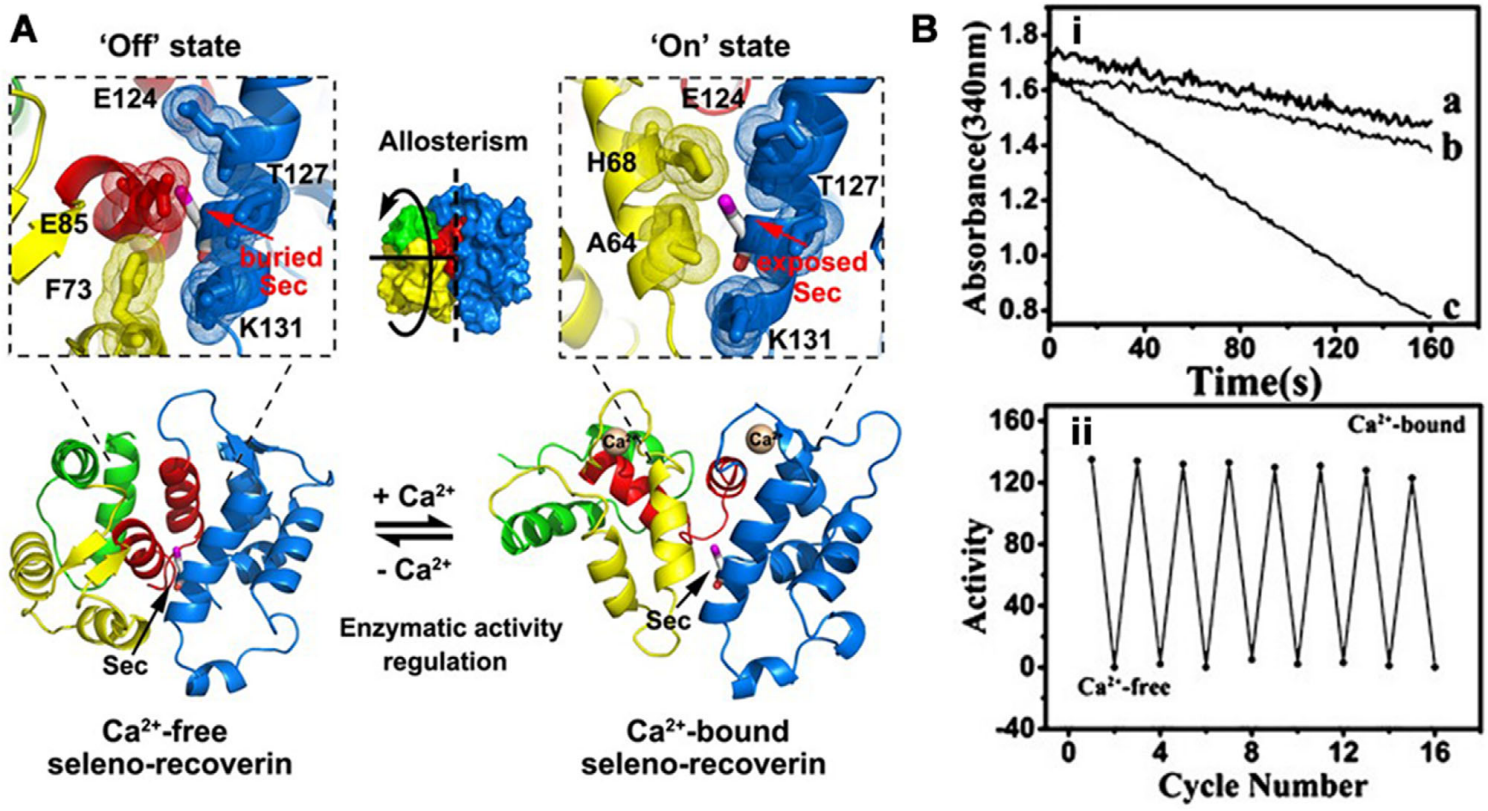
(A) Reversible switching of an artificial selenoenzyme based on the allosteric protein recoverin incorporated with selenocysteine as the active center. (B‐i) The significant difference between the catalytic curves of Ca^2+^‐free and Ca^2+^‐bound seleno‐recoverin‐Q50R. (B‐ii) Reversible switch of the selenoenzyme upon calcium ions. Adapted with permission.^[^ ^69^ ^]^ Copyright 2014, Wiley‐VCH

The incorporation of non‐natural amino acid residues also expands the application of this allosteric design strategy. Recently, a Ca^2+^‐responsive bioinspired antioxidant selenoenzyme was constructed by computational design and engineering of an allosteric protein scaffold,^[^ ^69^ ^]^ as the oxidative injury is usually coupled with high Ca^2+^ concentration in mitochondria. The active center selenocysteine was inserted into the allosterically variable site on the scaffold by site‐directed mutation and a cysteine‐auxotrophic system. This selenoenzyme can reduce excessive hydroperoxides into harmless hydroxyl compounds at the expense of glutathione and be switched on/off by Ca^2+^‐induced allosterism of the recoverin protein, as the active center is exposed in the Ca^2+^‐bound conformation and embedded in the Ca^2+^‐free conformation (Figure 2). In addition, the catalytic activity of this enzyme (140 μmol min^–1^ μmol^–1^) is in the same order of magnitude as that of natural glutathione peroxidase (GPx) in human plasma, and completely reversible after multiple Ca^2+^‐bound and Ca^2+^‐free cycles. Such a selenium‐containing system has also been utilized to construct an ATP‐switched antioxidant enzyme.^[^ ^70^ ^]^

### 2.2 Switchable synthetic catalysts

Synthetic allosteric catalysts not only allow for the development of economic catalysts for industrial applications but also provide access to regulating non‐natural reactions in which the activity/selectivity can be switched by the effector. Compared with large molecules such as proteins, synthesized molecules have a simple, stable, and controllable structure, and can be easily modified. Various functional groups afforded many stimulus‐responsive molecules that are sensitive to light, pH, metal coordination, redox, and mechanical forces. These allosteric molecules could conduct large geometrical changes upon external conditions, thus the catalytic group would undergo a dramatic transformation of the chemical environment by coordination, steric, electronic, and aggregation effects. Moreover, the combination of allosteric synthetic molecules with protein catalysts by chemical modification or genetic engineering also offers a critical strategy to develop smart biocatalysts which have both dynamic features and high efficiency. The hybridization of artificial molecular switches and natural enzymes have been applied in switchable glutathione S‐transferase and split‐luciferase complementation.^[^ ^71^ ^]^ It is critical to incorporate the synthetic switch at proper positions on the enzymes to achieve the switchable activity.

Azobenzene derivatives have been widely studied due to the large geometrical change between trans/cis isomers upon radiation.^[^ ^72^ ^]^ Depending on the size of the modified functional groups to the benzene rings, the light that triggers the conformational change could vary on the wavelength. The robust light‐responsive feature of azobenzene derivatives makes these synthetic allosteric molecules universal in many large‐scale systems, such as the core of dendrimers,^[^ ^73^ ^]^ molecular switch modified on proteins,^[^ ^71^ ^]^ and supramolecular assemblies. Besides, Branda's group reported the first case of a light‐driven chiral metal catalyst based on the framework of 1,2‐dithienylhexafluorocyclopentene.^[^ ^74^ ^]^ By introducing a chiral oxonate at the ortho positions of two thienyl groups, they synthesized a bisoxazoline chiral ligand, which can be reversibly switched between State I and State II by light. The author chose the cyclopropanation of styrene as the target reaction. In State I, the formation of intramolecular bidentate coordination metal catalysts afforded moderate enantioselectivity. While in State II the photo‐induced ring‐closure catalytic species formed monodentate coordination, resulting in almost racemic products.

**FIGURE 3:**
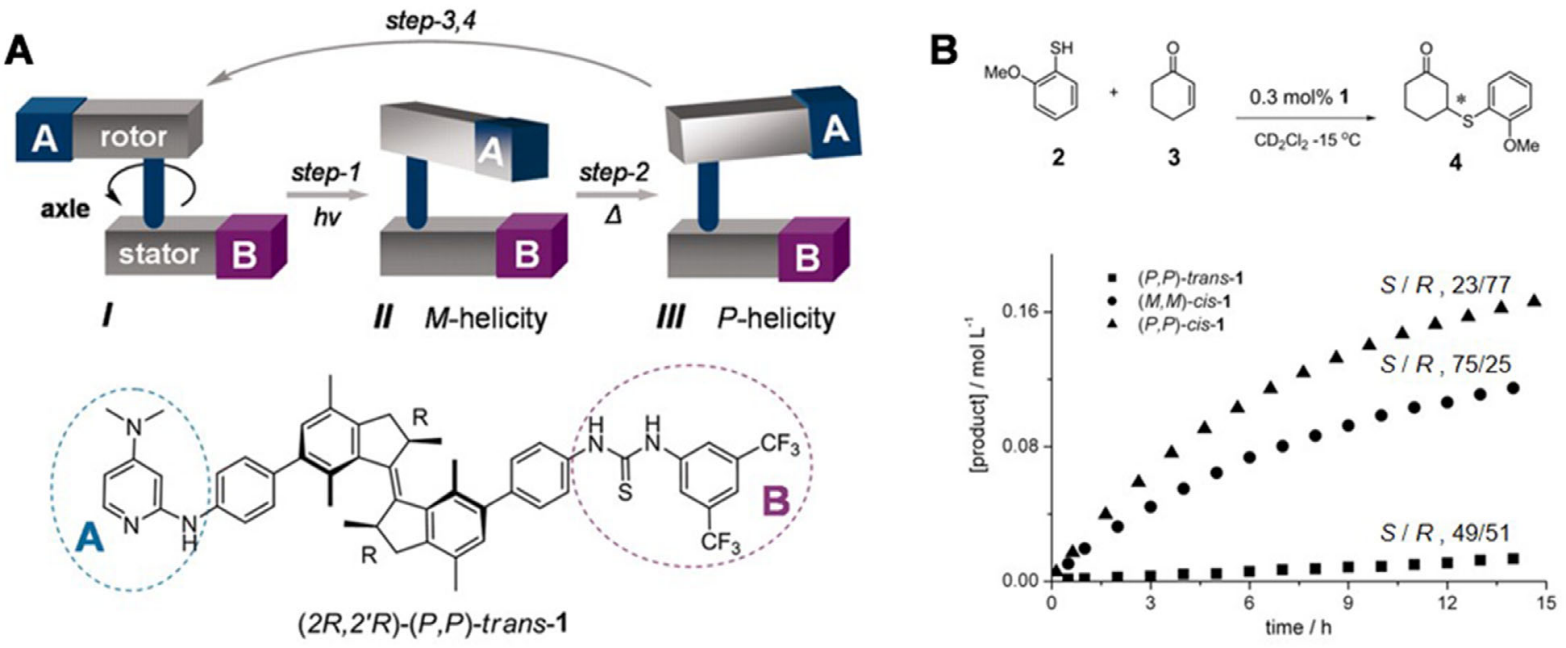
(A) Light‐driven molecular motor for asymmetric catalysis of Michael addition. (B) The relative rates and enantiomeric ratio of product formation for the three catalysts (*P*,*P*)‐*trans*‐**1**, (*M*,*M*)‐*cis*‐**1**, and (*P*,*P*)‐*cis*‐**1**. Adapted with permission.^[^ ^75^ ^]^ Copyright 2011, American Association for the Advancement of Science

Moreover, Feringa and coworkers develop a 360° rotation and four‐states photoswitchable molecular motor that could promote Michael additions (Figure 3).^[^ ^75^ ^]^ In different states, the yield of the catalytic reaction ranged from 7% to 83% with dramatically different stereoselectivity, showing the potential of synthetic allosteric molecules in asymmetric catalysis. Subsequently, they explored the universality of this light‐driven chiral molecular motor catalyst and extended its application to other asymmetric catalytic reactions. Based on the successful application of molecular motors in the asymmetric catalysis of small organic molecules, Feringa's team further expanded it to the field of metal catalysis.^[^ ^76^ ^]^ Recently, they developed the second‐generation molecular motor to construct a new artificial stimulus‐response chiral catalytic system.^[^ ^77^ ^]^ Unlike the first‐generation molecular motor, the second‐generation molecular motor has different metastable states during the rotation process. They introduced the biphenyldiol unit into the molecular switch and found that the chiral center could induce the helical chirality of the molecular switch. Meanwhile, under the irradiation of different wavelengths of light, the stable State I and its semi‐stable State II showed reversible transformation.^[^ ^78^ ^]^ With this chiral molecular switch as the ligand, in the asymmetric addition reaction of diethylzinc to benzaldehyde derivatives, the two‐state ligands (State I and State II) afforded opposite configuration, and the maximum difference in ee value reached 113%.

In 2017, Chen and Yu et al. reported a novel helical chiral switch catalyst containing a 4‐aminopyridine catalytic center.^[^ ^79^ ^]^ A pair of diastereoisomers with different helical chirality could be obtained by light/thermal stimulation. In the asymmetric Steglich rearrangement reaction of O‐acylazlactone, those isomers could obtain chiral quaternary carbon products with the opposite configuration in high yield and enantioselectivity [91% ee and 94% ee, respectively], which is currently the best photo‐responsive chiral catalyst.

In addition to small molecular switches, many polymers exhibit allosteric behavior near the lower critical solution temperature (LCST). Poly(*N*‐isopropylacrylamide) (PNIPAM) is extensively studied as a typical temperature‐responsive polymer scaffold with an LCST of 32°C, which is close to the temperature under physiological conditions. However, using PAMAM as a scaffold for the construction of switchable catalysts remains rare. Temperature‐responsive polymers were usually applied as phase separation agents in multicomponent systems to achieve temperature‐responsive catalysis. For instance, Lu and coworkers developed a yolk‐shell system in which a single Au nanoparticle was encapsulated in a thermosensitive polystyrene‐poly(*N*‐isopropylacrylamide) shell.^[^ ^80^ ^]^ The porosity and the hydrophobicity of the polymer shell could be dramatically changed by temperature, which acted as a trigger to enhance the catalytic selectivity for given substrates. Recently, another thermoresponsive polymer tannin‐aminopropyltriethoxysilane was coated on the surface of halloysite nanotubes together with PNIPAM to immobilize Au nanoparticles.^[^ ^81^ ^]^ These multicomponent systems with rigid nanostructures showed significant thermoresponsive catalytic performance in the reduction of 4‐nitrophenol, and the highest activity was acquired at the LCST of 45°C.

## 3 ALLOSTERIC CATALYSTS BASED ON SYNTHETIC SUPRAMOLECULAR COMPLEXES

Reversibility is one of the most significant features of supramolecular interactions, which have been widely utilized to design switchable catalysts. The reversible binding and dissociation of weak interactions have bred the allosteric behaviors of proteins. In this section, we expanded the meaning of “allosteric” to describe the conformational change of a supramolecular system rather than one single molecule. Undoubtedly, this “change” caused by the allosteric behavior of supramolecular systems offers the opportunity to control the catalytic activity. Specifically, the different binding states between the host molecule and the guest molecule could exhibit opposite chemical environments, which would lead to a notable change in stability, solubility, and catalytic activity. In this case, the external stimulus that triggers the shift between two binding states of the supramolecular system would act as the allosteric effector. With the development of supramolecular chemistry, many host‐guest systems have been extensively studied. It is a research hotspot to incorporate catalytic groups into these systems and investigate the effects of confined space and dynamic molecular recognition on catalytic reactions.

### 3.1 Switchable catalytic host‐guest systems

**FIGURE 4:**
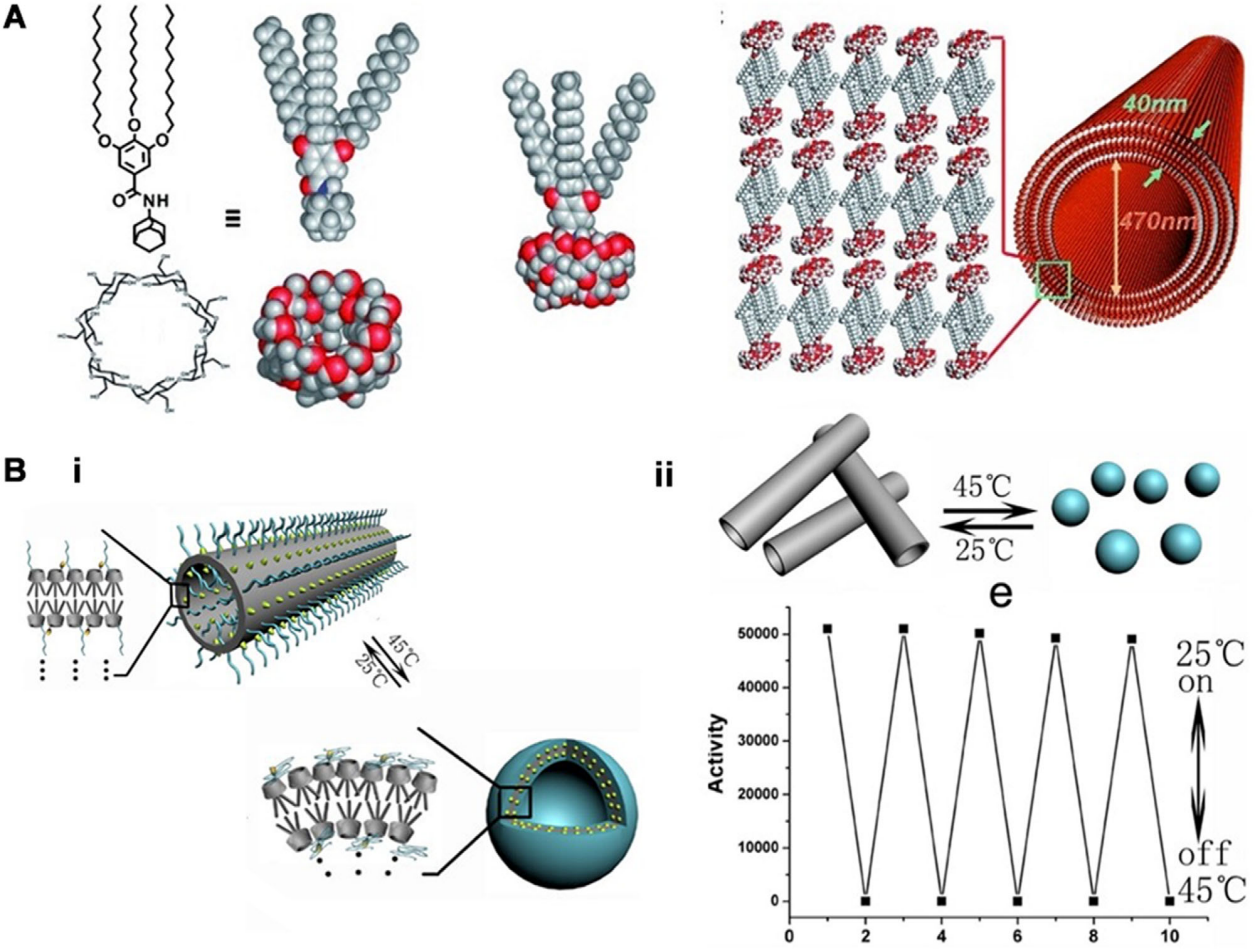
(A) Catalytic assemblies constructed by tellurium‐containing supra‐amphiphiles. Adapted with permission.^[^ ^82^ ^]^ Copyright 2010, Wiley‐VCH. (B‐i) The morphology could be regulated between nanotubes and nanospheres. (B‐ii) A reversible switch of peroxidase activity by tube‐sphere transformation at two different temperatures. Adapted with permission.^[^ ^83^ ^]^ Copyright 2014, the American Chemical Society

Cyclodextrins have been extensively studied due to their capability to reversibly encapsulate hydrophobic guest molecules in the bowl‐like cavity. Also, cyclodextrins are easy to be modified with catalytic functional groups, which makes them promising candidates as artificial enzyme scaffolds. A tellurium atom was incorporated into cyclodextrin to endow the host molecule with antioxidant activity, and the host‐guest interaction of cyclodextrin and adamantane afforded a tellurium‐containing supramolecular amphiphilic complex, which used the cyclodextrin as a polar head group and the long chains of the guest molecules as a hydrophobic tail (Figure 4A).^[^ ^82^ ^]^ Then the amphiphilic complex could assemble into supramolecular nanotubes by stacking in water. Inspired by the fact that natural GPx contains positively charged residues to enhance the enzyme‐substrate recognition, the nanotube was further modified with the guanidine group to improve the antioxidant activity. Due to the synergistic multiple catalytic sites and large superficial area, the nanotubes were four orders of magnitude more active than diphenyl diselenide. Based on the above‐mentioned assembled nanotubes, PNIPAM was linked to the Tellurium‐containing cyclodextrin by ATRP polymerization and click chemistry methods to get the temperature response characteristics (Figure 4B).^[^ ^83^ ^]^ Through temperature control, a reversible transformation between nanotubes and vesicles was observed. At low temperatures, the tellurium atom, which is the active center of the enzyme, is exposed outside the vesicle, so the vesicle exhibits very high catalytic activity. At high temperatures, due to the hydrophobicity change of the PNIPAM group, the nanotube transforms to a more favorable vesicle structure. Meanwhile, the tellurium active center is embedded in the vesicle, resulting in the activity loss of the enzyme. In this way, a temperature‐responsive nanozyme was constructed by varying the dynamic assembly morphologies.

### 3.2 Switchable catalytic rotaxane systems

**FIGURE 5:**
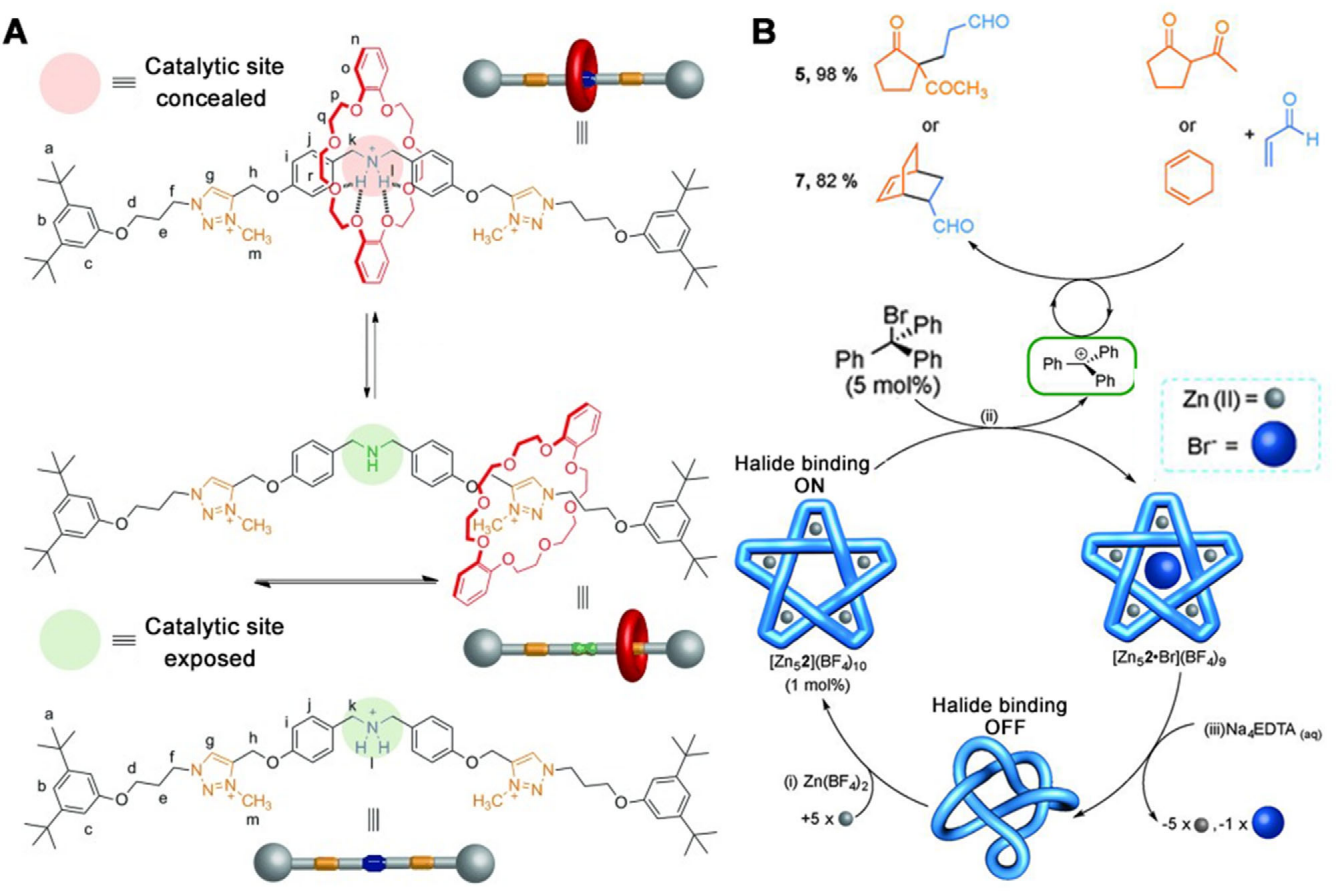
(A) Acid‐base switching of a rotaxane catalyst by alternating the preferential binding site of the macrocycle. Adapted with permission.^[^ ^84^ ^]^ Copyright 2012, Wiley‐VCH. (B) Switchable catalysis of Michael addition and Diels–Alder reactions by in situ generations of a trityl cation via bromide abstraction using Zn(II)–pentafoil knot [Zn_5_2](BF_4_)_10_. Adapted with permission.^[^ ^86^ ^]^ Copyright 2016, The Nature Publishing Group

The sliding of macrocycles along the axle of rotaxane systems is highly controllable and reversible. That is, if two or more binding sites for the macrocycle are modified on the thread molecule, the sliding macrocycle would prefer the one with a higher binding constant at specific external stimuli such as pH, temperature, and solvents. For instance, a pH‐switched rotaxane catalyst was developed by Leigh and coworkers to control the Michael addition of an aliphatic thiol to *trans*‐cinnamaldehyde (Figure 5A).^[^ ^84^ ^]^ The dibenzylamine group, which acted as the catalytic center, was modified on the axle molecule together with two triazolium rings as alternative binding sites. When the dibenzylamine is protonated, the macrocycle binds to the ammonium group so that the catalytic amine is blocked by the macrocycle to turn off the catalysis. However, if the dibenzylamine of the rotaxane is not protonated, the macrocycle would bind the triazolium groups, then the dibenzylamine group was revealed to turn on the catalytic activity. By this means, the activity can be turned “on” and “off” upon pH change. The rotaxane catalyst could catalyze the β‐functionalization of carbonyl compounds with C or S‐nucleophiles through iminium activation with remarkable conversions (95–98%). Also, the catalytic rotaxane could control the rate of nucleophilic addition or substitution reactions via examining catalysis with a better switching effect. Additionally, the “on‐state” of the rotaxane was able to promote tandem iminium‐enamine reaction sequences and the Diels–Alder reaction of dienals through a trienamine activation mode. Furthermore, Leigh's team added a benzene ring group near the catalytic center, which is equivalent to introducing an asymmetric factor, enabling the smart enzyme system to perform asymmetric catalysis with high stereoselectivity.^[^ ^85^ ^]^ Through experienced synthesis technology, Leigh and coworkers also developed a molecular pentafoil knot that allosterically initiates or regulates catalyzed chemical reactions by controlling the in situ generations of a carbocation formed through the knot‐promoted cleavage of a carbon‐halogen bond (Figure 5B).^[^ ^86^ ^]^ A molecular knot is a mechanically interlocking molecular structure similar to a knot. The two most common naturally occurring molecular knots in nature exist in protein and DNA. This is the first time to achieve the removal and re‐coordination of coordination metals in the junction, and the impact of metal coordination on the catalytic performance of artificially synthesized molecular knot was demonstrated.

### 3.3 Switchable catalytic coordination compounds

Mirkin et al. have reported a tetrametallic complex prepared via the weak‐link approach to supramolecular chemistry, that behaves as an allosteric catalyst for the ring‐opening of cyclohexene oxide.^[^ ^87^ ^]^ The allosteric effect is achieved through a modification of catalytic activity via the binding of a CO molecule and a Clion to each Rh(I) metal at the structure control sites, distal from the Cr (III) metal centers at functional sites within the same macrocyclic assembly. When the catalyst activity is in a closed state, the initial rate kinetics for the ring‐opening of cyclohexene oxide could be reduced to 30%. Moreover, the selectivity and activity of this supramolecular catalyst in the asymmetric ring‐opening of cyclohexene oxide were enhanced compared to a Cr(III)‐salen monomeric analog. As the first demonstration of an allosteric catalyst made possible through supramolecular coordination chemistry, it shows how one can use the weak‐link approach to design and build supramolecular complexes with a unique function. One year later, Mirkin and coworkers further develop another “molecular tweezer” by utilizing the Rh coordination as the structural control site at the center of the designed catalyst.^[^ ^88^ ^]^ When the Rh center coordinated with CO and Cl ligands, the synthetic catalyst exhibited a linear form. However, the intramolecular coordination between Rh could replace the CO and Cl ligand and bend the catalyst into a tweezer form. This allosteric transformation also led to a significant impact on the catalytic performance for the ring‐opening of cyclohexene oxide.

**FIGURE 6:**
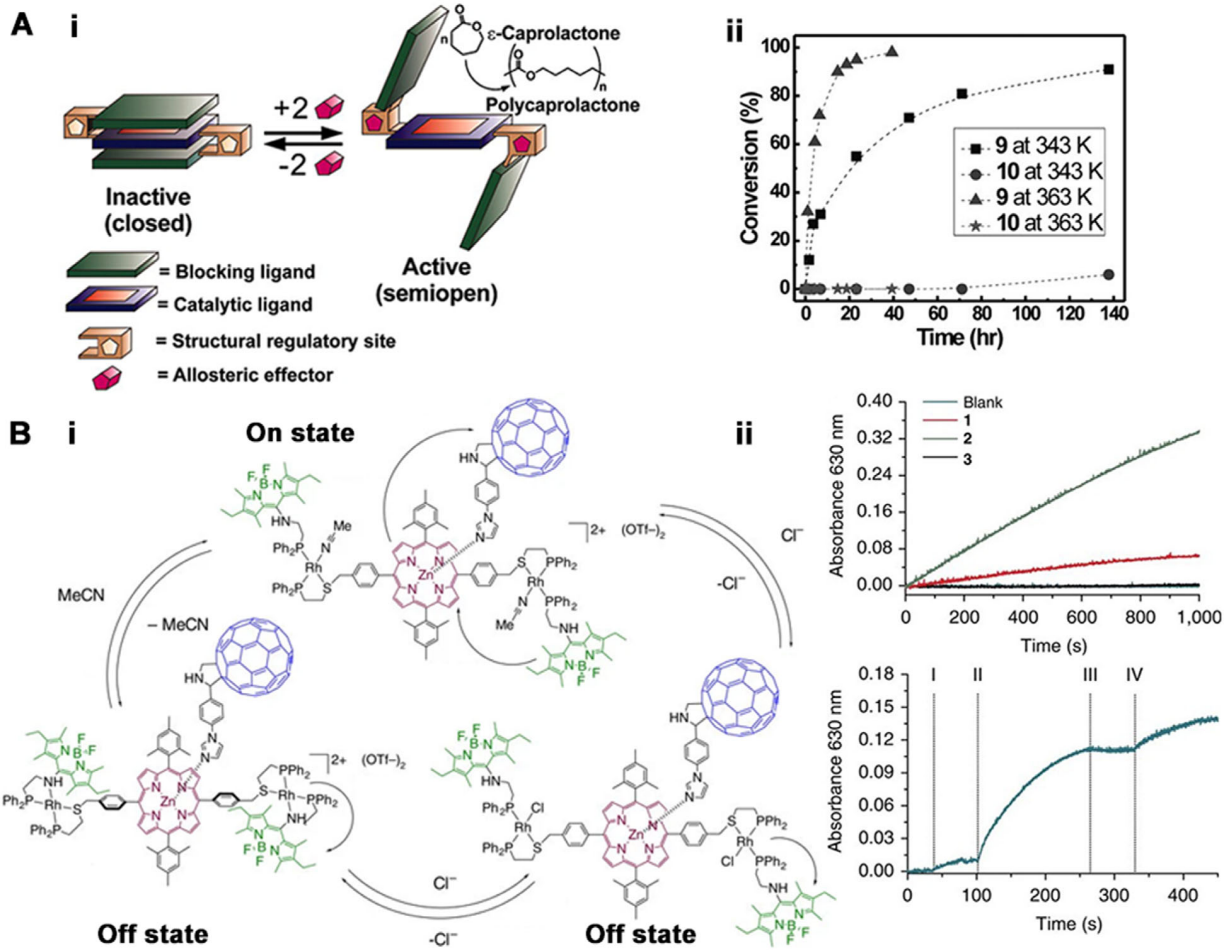
(A‐i) A model of an allosteric supramolecular triple‐layer complex for the regulation of the catalytic living polymerization of є‐caprolactone. (A‐ii) catalytic activities of semiopen and closed states in the polymerization of ε‐caprolactone. Adapted with permission.^[^ ^89^ ^]^ Copyright 2010, American Association for the Advancement of Science. (B‐i) Allosteric regulation of a light‐harvesting antenna/reaction center mimic. (B‐ii) Catalytic reduction of methyl viologen in the presence of (1–3)‐ImC_60_. (I) turning excitation source on, (II) addition of acetonitrile, (III) addition of chloride, and (IV) addition of Tl. Adapted under the terms of the Creative Commons CC BY license.^[^ ^90^ ^]^ Copyright 2015, The Nature Publishing Group

Later, Mirkin and coworkers further developed an allosteric supramolecular structure in which a monometallic catalytic site has been buried in the middle layer of a triple‐layer complex (Figure 6A).^[^ ^89^ ^]^ The triple‐layer complex (TLC) is composed of two transition metal nodes, two chemically inert blocking exterior layers, and a single catalytically active interior Al(III)‐salen complex, which can act as a ring‐opening polymerization catalyst with є‐caprolactone (є‐CL) as the substrate. This complex could be reversibly activated and deactivated with an assemble/disassemble trilayer structure through acetonitrile and Cl^–^ abstracting agent (e.g., NaBArF and LiB(C_6_F_5_)_4_∙Et_2_O). In the activated state with a semi‐open structure, the conversion rate of 100% can be achieved within 40 h of catalysis, while the conversion rate in the deactivated state (with a fully closed triple‐layer structure) is almost zero within 100 h.

Inspired by photosynthetic machinery, the same group developed the first light‐collecting antenna/reaction center simulation in 2015, which could be adjusted by using a coordination frame incorporating antenna hemilabile ligands and assembled by a high‐throughput modular strategy (Figure 6B).^[^ ^90^ ^]^ The light‐collecting antenna/reaction center simulation model is composed of Bodipy, porphyrin, and fullerene units. Rh(I) coordination is introduced into the system, and its interaction with allosteric coordination effectors can be used to disrupt the electrochemical landscape of the framework as remarkably mild and redox‐inactive inputs, resulting in the regulation of the photoredox catalytic activity of the photosynthetic mimic reversibly and in situ. The volume conversion ratio between the active and inactive states of this allosteric photoredox catalyst is up to 39‐fold in terms of energy transfer efficiency.

## 4 ALLOSTERIC CATALYSTS BASED ON SELF‐ASSEMBLY

Natural enzymes usually prefer to work together in the form of oligomers in sophisticated biological processes. The critical and complicated regulatory conjunctions in metabolic pathways are often controlled by a multi‐component complex, which includes a variety of coordinated enzymes, regulatory factors, and structural motifs. These protein complexes as the supervisors of biological activities have well‐organized nanostructures and specific functions. Generally, the self‐assembly of natural enzymes brings higher stability, better coordination between enzymes, and dynamic feature in favor of metabolic regulation. The assembly and dissociation of components not only are related to significant physiological indexes but also control enzymatic activity smoothly and efficiently. If the dynamic binding‐dissociation process of one host‐guest system could be utilized to control the catalytic activity, reversible supramolecular self‐assemblies at the nanoscale could also exhibit “allosteric” morphologies to convey the function of switchable catalysis.

### 4.1 Switchable catalytic protein assemblies

Studies on the combination of the catalyst design and protein self‐assembly have emerged, and one key issue in this field is that natural enzymes would suffer a significant loss of catalytic activities once they were assembled into an artificially designed protein assembly. Because the catalytic sites of these enzymes are formed after millions of years of evolution, and the tiny structural fluctuations caused by the assembly process could severely affect the catalytic activity. Besides, it is quite difficult to design new connections between protein building blocks from scratch. In this case, the redesign of new catalytic sites on the existing protein assembly scaffolds would be more promising to develop switchable biocatalysts. The intrinsic properties of protein building blocks (e.g., thermal stability, allosterism) would be reflected in the entire assemblies, which extend the application of these catalysts.

**FIGURE 7:**
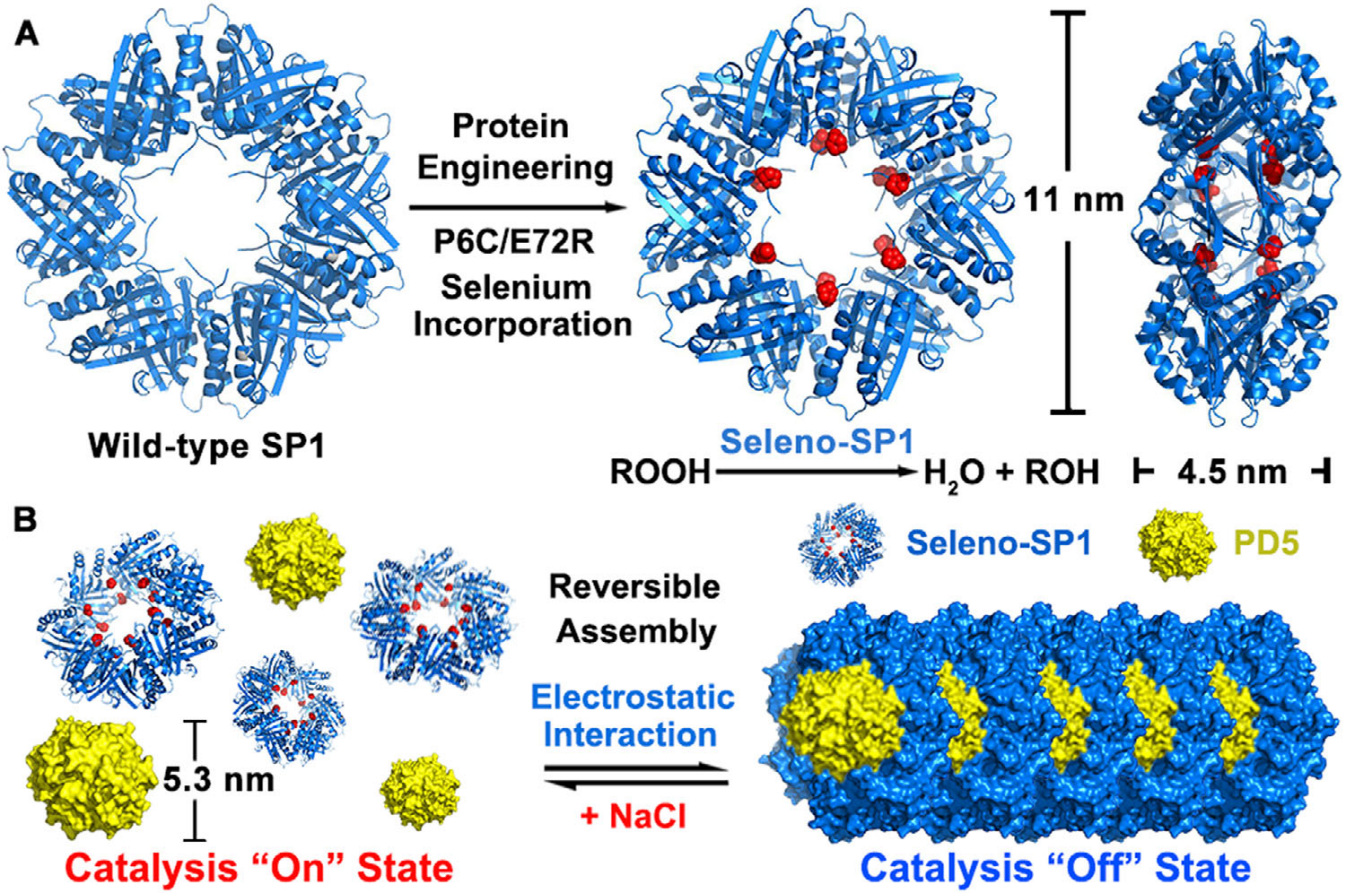
Controllable antioxidant system based on the reversible self‐assembly of SP1 with PD5. Adapted with permission.^[^ ^94^ ^]^ Copyright 2020, The American Chemical Society

Supramolecular interactions that drive the self‐assembly process could be robust and reversible, which offers protein assemblies a great advantage in the design of switchable catalysts. For instance, SP1 is a ring‐like homo‐oligomeric protein, consisting of twelve subunits that are bound together via hydrophobic interactions forming two stacked hexameric rings.^[^ ^91^ ^]^ Due to the intrinsic stable feature of SP1, the artificial selenoenzyme based on the SP1 scaffold exhibited high thermal stability that it remains active under 85°C for days or 37°C for months.^[^ ^92^ ^]^ Also, SP1 as a negatively charged protein building block could further assemble with positively charged particles by electrostatic interactions.^[^ ^93^ ^]^ Based on the dynamically controllable protein assembly, the selenium‐modified protein SP1 (Seleno‐SP1) was assembled with the dendrimer PD5 into a robust and controllable nanowire.^[^ ^94^ ^]^ The trick in this protein‐dendrimer hybrid assembly is that the selenium catalytic sites locate on the plane of SP1 disks, which allows the control of embedding/revealing catalytic sites upon the repeated reversible assembly and dissociation process, respectively (Figure 7). This system not only showed high activity that rivals that of natural GPx but also exhibited a regulatory mechanism by ionic strength, as the ionic strength has a significant impact on the electrostatic interaction between Seleno‐SP1 and PD5. The blocking and restoring efficiencies of the catalytic activity of the system reached 95.5% and 98.7%, and the switching effect can last for multiple cycles, showing only a limited attenuation of activity. In addition, the morphological characterization of Seleno‐SP1/PD5 with different concentrations of NaCl proved that the change of its catalytic activity highly corresponds to the reversible assembly and dissociation behavior. This research provided a new method for building a switchable antioxidant system and establishes a bridge between the dynamic structure and controllable functions of protein assemblies.

### 4.2 Switchable catalytic peptide assemblies

Although natural enzymes have excellent catalytic efficiency and substrate selectivity, they are accompanied by relatively expensive production costs and poor stability of enzymes. Generally, enzymes essentially have one or more polypeptide chains undergoing complicated folding and various modifications to finally come into function, which requires strict and precise regulation and conformational control, significantly limiting the wide application of enzymes in industry. From this point of view, if the structure of the enzyme is simplified and reinforced, lower‐cost catalysts could be developed to achieve the purpose of practical application. As building blocks of proteins, peptides have been developed to catalyze many chemical reactions, despite their relatively short sequences and simple structures. Also, the incorporation of synthetic groups and non‐natural amino acids further extends the catalytic scope of the peptide‐based catalysts. In addition, the self‐assembly of functional peptides into well‐organized complexes, just like another kind of “evolution,” offers an inspiring perspective to construct robust and efficient catalytic biomaterials.

The development of self‐assembling peptide‐based catalysts has been widely applied in many catalytic reactions such as hydrolysis and Michael additions. However, it is challenging to enrich the catalytic scope and regulation of peptide‐based catalysts. To address this issue, the selenium‐containing peptide derivative Fmoc‐phenylalanine‐selenide was developed and self‐assembled into nanospheres or nanotubes according to the redox environment in the system.^[^ ^95^ ^]^ This is because selenium can be reduced and oxidized by glutathione and peroxide, respectively, so that it can be converted between selenide and selenoxide. According to different oxidation‐reduction states of the selenium element, the assembly morphology of the selenium‐containing peptide derivative is changed. Moreover, the inherent hydrophobicity and aromaticity of the Fmoc moiety can promote the association of building blocks. When selenium existed in the form of selenoxide, it would be exposed on the surface of the assembly, so that the system showed GPx activity. On the contrary, when selenium existed in the form of selenide, it would be embedded in the assembly could not exhibit the antioxidant function. In this way, the selenium‐containing peptide derivative can intelligently perform its function according to the oxidative pressure of the external environment and maintain the amount of hydroperoxide in the system at a specific level. The core of this work is the introduction of a new catalytic center and catalytic function, and the utilization of the dynamic and switchable properties of peptide assemblies to regulate the catalytic function.

Given the example above, one would expect that there must be a critical relationship between the ordered arrangement of supramolecular structures and the catalytic activity. If the catalytic cavity of the enzyme‐like peptide assembly is similar to that of natural enzymes, which contains not only the catalytic site but also essential groups to enhance the enzyme‐substrate binding, it is anticipated that the catalytic activity of peptide‐based catalysts could be improved. To investigate whether this strategy works, a binary peptide assembly system containing histidine (NH_2_‐HSGQQKFQFQFEQQ‐Am) and arginine (NH_2_‐RSGQQKFQFQFEQQ‐Am) was developed to form amyloid‐like nanofibers with sol‐gel transformation.^[^ ^96^ ^]^ Enzymology experiments showed that the Michaelis constant of the binary peptide assembly was significantly lower than that of the monolithic assembly formed by the histidine‐containing peptide alone, which proved that the substrate‐binding ability provided by arginine and the catalytic center formed a synergistic effect. As a single peptide molecule could not provide such complex conformation similar to the catalytic center of a natural enzyme, but the self‐assembly strategy can benefit the binding between peptide‐based catalysts and the substrate, stabilizing the transition state and maintaining higher catalytic efficiency.

**FIGURE 8:**
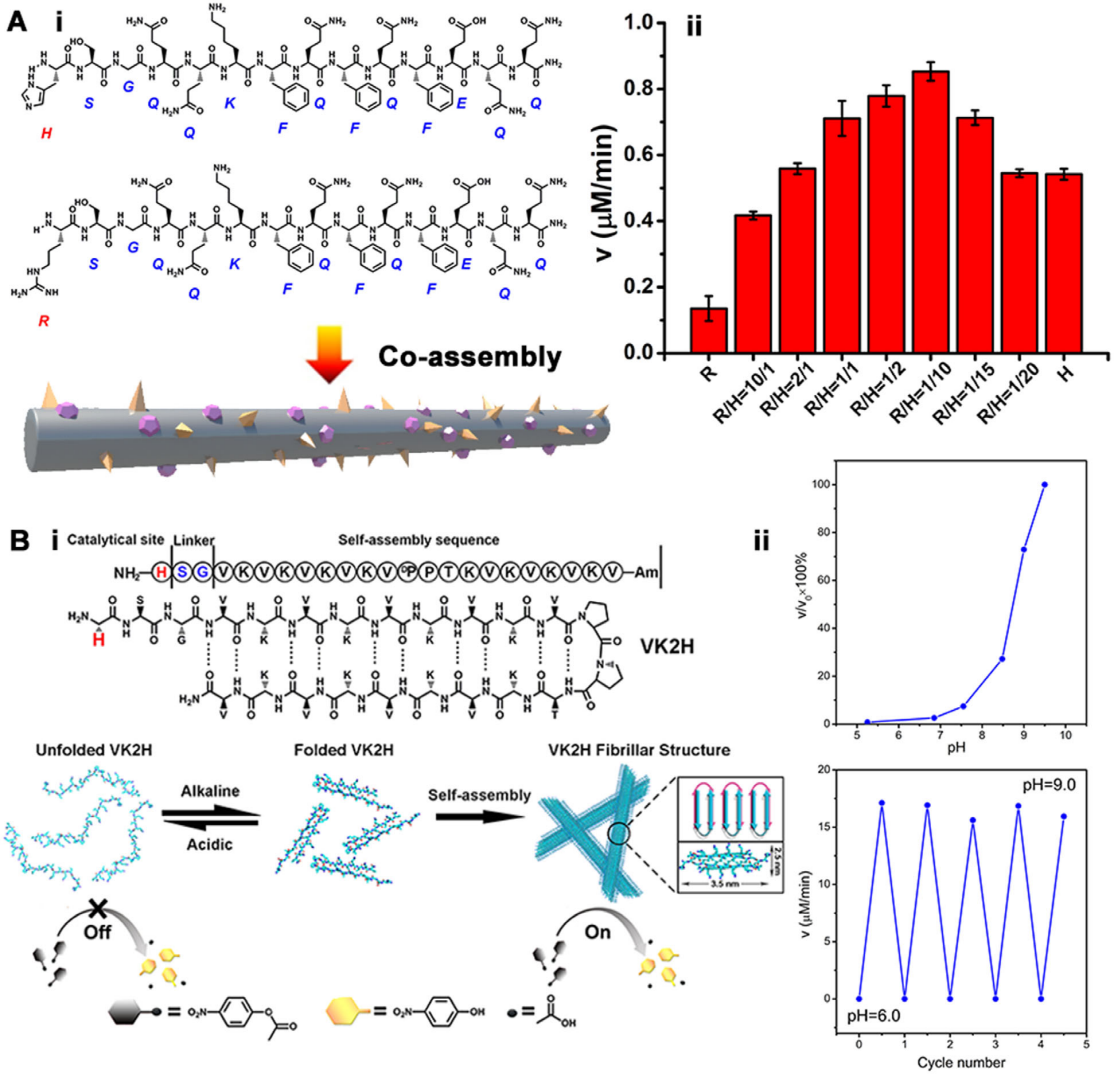
(A‐i) The scheme of Q11H and Q11R co‐assembly to form amyloid‐like nanofibers. (A‐ii) Catalytic reaction rates for hydrolysis of pNPA vs molar ratio of Q11R to Q11H. Adapted with permission.^[^ ^96^ ^]^ Copyright 2014, The American Chemical Society. (B‐i) The pH‐switched artificial hydrolase based on conformational change of VK2H and the cartoon structure of the bilayer. (B‐ii) Catalytic rate of VK2H nanofibers at different pH versus the initial catalytic rate of VK2H fibrils at pH 9.5 and the on‐off switch of catalytic activity of VK2H peptide by a change in pH between 6.0 and 9.0. Adapted with permission.^[^ ^97^ ^]^ Copyright 2017, Wiley‐VCH

Since the supramolecular structure of peptide assemblies plays a critical role in the catalytic activity, incorporation stimulus‐responsive groups into the peptide build blocks would provide a new strategy to design switchable catalysts based on controllable peptide assemblies. Ulijn and coworkers modified a hydrolytic active histidine on the terminus of a pH‐responsive peptide (Figure 8).^[^ ^97^ ^]^ At neutral pH, the peptide adopted a random coil configuration. While the pH increased to 9, the peptide formed a β‐sheet configuration. The accompanying activity change was also as high as 100 folds. Moreover, the hydrolytic activity was highly reversible, showing limited attenuation after five on/off cycles. This work proved that the dynamic self‐assembly process is a robust and reversible allosteric model, exhibiting great potential in the design of switchable catalysts.

## 5 CONCLUDING REMARKS AND PROSPECTS

The strict regulation of natural enzymes has inspired scientists in the development of artificial switchable catalysts to control the progress of chemical reactions. Among the naturally occurring mechanisms for activity manipulation, allosterism plays a critical role in the control of complex biological transformations. Ranging from allosteric proteins, stimulus‐responsive molecules, supramolecular complexes, and self‐assemblies, many impressive works have been developed by incorporating catalytically active groups in these allosteric scaffolds. Typical examples of allosteric catalysts mentioned above are summarized in Table 1. Despite the drawbacks such as narrow substrate scope and elaborate preparation, this strategy facilitates high efficiency and precise regulation, showing great opportunity to challenge nature's creation.

**TABLE 1:** The summary of allosteric catalysts constructed by different scaffolds

**Classification**	**Building scaffold**	**Catalytic reaction**	**Switch trigger**	**Design strategy**	**Ref**.
Single molecule	CaM‐F92E	Kemp elimination	Ca^2+^	Genetic engineering and directed evolution	67
	Seleno‐recoverin‐Q50R	Peroxide reduction	Ca^2+^	Non‐natural amino acid and computational design	69
	Molecular motor	Michael addition	Light	Photo‐induced allosteric group	75
Synthetic supramolecular complex	Cyclodextrin/PNIPAM complex	Peroxide reduction	Temperature	Temperature‐induced allosteric polymer	83
	Rotaxane system	Michael addition	pH	pH‐induced protonation of macrocycle binding sites	84
	Coordination compound	ring‐opening polymerization	Light	Photo‐induced allosteric group	89
Self‐assembly	Seleno‐SP1/PD5 assembly	Peroxide reduction	Ionic strength	Embedding/revealing the active site by electrostatic‐driven assembly	94
	Q11 peptide assembly	Hydrolysis	Ionic strength	Embedding/revealing the active site by salt‐driven assembly	97

In our opinion, the future of designing switchable bioinspired enzymes should lie in the following topics: (i) Expanding the catalytic scope of bioinspired enzyme models. The successful designs are mainly concentrated on simple reactions such as oxidoreduction and deprotonation, while switchable catalysts for sophisticated and multistep organic reactions remain rare. (ii) Building novel allosteric scaffolds for switchable biocatalysts. The technology for connecting different protein domains by gene insertion and swapping has been developed, and new synthetic allosteric molecules will emerge, providing more scaffolds for the design of switchable catalysts. (iii) Developing in vivo applications for switchable catalysts. A successful bioinspired enzyme should be capable of its natural counterpart's biological function, which is rarely demonstrated in vivo. To meet practical demand, it is essential to construct switchable bioinspired catalysts that can make a difference at the cellular and macroscopical level. In the end, we hope that this review of switchable bioinspired catalysts may inspire fresh ideas and accelerate the pace of further explorations.

## CONFLICT OF INTEREST

Xue Xue is a member of the *Exploration* editorial board. The authors declare no conflict of interest.
